# An International Federal Hyperledger Fabric Verification Framework for Digital COVID-19 Vaccine Passport

**DOI:** 10.3390/healthcare10101950

**Published:** 2022-10-06

**Authors:** Dong-Her Shih, Pai-Ling Shih, Ting-Wei Wu, Shu-Huai Liang, Ming-Hung Shih

**Affiliations:** 1 Department of Information Management, National Yunlin University of Science and Technology, Douliu 64002, Taiwan; 2 Department of Information Management, National Chung Cheng University, Chiayi 621301, Taiwan; 3 Department of Electrical and Computer Engineering, Iowa State University, 2520 Osborn Drive, Ames, IA 50011, USA

**Keywords:** blockchain, Hyperledger Fabric, COVID-19, digital vaccine passport (DVP), federated identity management

## Abstract

The COVID-19 virus has been spreading worldwide on a large scale since 2019, and the most effective way to prevent COVID-19 is to vaccinate. In order to prove that vaccination has been administered to allow access to different areas, paper vaccine passports are produced. However, paper vaccine passport records are vulnerable to counterfeiting or abuse. Previous research has suggested that issuing certificates digitally is an easier way to verify them. This study used the consortium blockchain based on Hyperledger Fabric to upload the digital vaccine passport (DVP) to the blockchain network. In order to enable collaboration across multiple systems, networks, and organizations in different trust realms. Federated Identity Management is considered a promising approach to facilitate secure resource sharing between collaborating partners. Therefore, the international federal identity management architecture proposed in this study enables inspectors in any country to verify the authenticity of the DVP of incoming passengers using the consortium blockchain. Through practical construction, the international federal Hyperledger verification framework for the DVP proposed in this study has shown the feasibility of issuing a global DVP in safety analysis and efficacy testing.

## 1. Introduction

The discovery of COVID-19 in 2019 caused the most significant disease infection in human history. Border personnel in various countries are more stringent than before in the entry qualification examination, and passengers also need to present health certificates to prove that they are not infected when entering public places in China [1]. As of 13 June 2022, COVID-19 has infected more than 500 million people and caused more than 6 million deaths globally, according to publicly available information released by the World Health Organization (WHO) [2]. Some patients’ main symptoms of COVID-19 are fever, cough, shortness of breath, and diarrhea. The main problem is that symptoms of the disease usually appear after two to 14 days if a person is infected. This period is called the incubation period; the average incubation period is about five days. An infected person can infect many healthy people during the incubation period, which is why COVID-19 has spread so quickly [3]. Governments of all countries hope to meet the COVID-19 resistance and reduce the risk of disease among their citizens while the vaccination rate is widespread. As knowledge and understanding of COVID-19 continues to evolve, developing appropriate response plans for the pandemic is considered one of the most effective ways to control the spread of the virus. Pang et al. [4] proposed a federated learning solution for COVID-19 response plan management, and the research results showed great potential in building intelligent models for new infectious diseases. Waggoner et al. [5] wanted to understand how people talked about the COVID-19 pandemic in social networks and thought that future studies like this could also delve deeper into how individual, global, and descriptive structures were found. With the outbreak of COVID-19, online learning has gradually become a mainstream way of learning. Agarwal et al. [6] found that e-learning has the potential to reduce carbon emissions, which has a beneficial impact on the environment. However, mental health is affected, as e-learning can lead to self-isolation and decreased academic performance leading to anxiety and depression. As a result of using electronic devices for studying, the eyes and neck muscles may be strained, with detrimental effects on physical health. Therefore, it is urgent to create a vaccine passport to prove that the vaccine has been administered [7]. This vaccine passport should have an anti-counterfeiting function, be easy to publish, easy to use, easy to verify authenticity, easy to authenticate, and easy to protect with regard to personal information in order to to expand the possibility of system popularization [8]. The Transportation Security Administration (TSA) of the USA [9] requires international travelers to have received two doses of the COVID-19 vaccine to be eligible for entry, so they must provide proof that they have been fully vaccinated before entering the United States. According to [8,10], traditional paper health certificates are easily falsified and tampered with, so recording and preventing record tampering by digital means is necessary.

Xu et al. [11] proposed that vaccine certification documents may be difficult to be widely used due to the privacy issues of the traceability of public tests. Chelladurai & Pandian [12] suggested that a tamper-proof capability should be added to the system, and data should be encrypted and adequately stored to provide a perfect medical personal health record system. Fatokun et al. [13] reviewed the past literature and concluded that data thieves may take advantage of patients’ personal privacy data for profit. Therefore, it is recommended that digital health record systems should be equipped with high security and privacy protection. Past studies have proved that blockchain technology has advantages in generating identity certificates [14]. The hash function ensures that unnecessary public data remain secret and cannot be modified to ensure that data cannot be falsified or arbitrarily altered. The traceable property ensures that every record can find the source of publication. Public and private keys and digital signatures ensure that no one can impersonate the identity of someone else, and decentralization ensures that a single point of failure does not bring the system down. Implementing digital vaccine certification can eliminate the shortcomings of traditional and identity certification shortcomings, which scholars have revealed in the past. Liu et al. [15] believe that when multiple different service providers need to provide services to the same user, federated identity management (FIM) architecture can reduce the trouble of digital identity management and improve security.

Due to the shortcomings of traditional paper identification in the past [14,16], there is a need for verifiable digital certificates to verify vaccine administration records. The digital record of vaccine administration is uploaded to the Internet via blockchain technology, and inspectors can access the record through an application. In addition, the digital vaccine passport generated from the blockchain can effectively prevent certificates from being forged and records from being tampered with, and all records can be traced back to the source to achieve immutable modification and traceability. If the federated identity management architecture is used, identity verification can be carried out among different service providers across the organization, and authentication data exchange can be achieved across the organization. However, the transnational digital vaccine certification through blockchain technology requires considerable scale and storage space, which is a noteworthy part in terms of cost [17].

To sum up, this study hopes to achieve the following objectives:

1. This study aims to develop a solution that provides privacy protection, anti-tampering, anti-identity theft, and proof of vaccination administration.
2. The screening results of all citizens will be uploaded to the blockchain through blockchain technology, and the health status of a fixed time will be recorded through the blockchain to make a DVP.
3. By introducing an international federal identity management framework, different countries can verify that their citizens have received vaccination certificates before entering the country.
4. The uploading and querying of transnational and inter-organizational vaccine administration records and verification of transnational vaccine administration records.
5. Through the international Hyperledger Fabric architecture, a stable and latency-reducing system flow of cross-border digital vaccine distribution and verification is established.

Section 2 of the study is the preliminary literature review, Section 3 is the verification framework of DVP, Section 4 includes the discussion, and Section 5 is the conclusion of the paper.

## 2. Preliminary

### 2.1. Hyperledger Fabric

Hyperledger Fabric is a modular, open source architecture blockchain platform developed by IBM. Fabric is a blockchain based on alliance chain [18]. In addition to the technical features of the original blockchain, Fabric requires a smart contract called Chaincode to enable peer-to-peer transactions within the blockchain network [19]. Chaincode can be used as a peer-to-peer interaction channel and interface to write transaction results into the ledger. Chaincode also supports rolling version upgrades and asymmetric version operations [18]. The consensus mechanism in Fabric adopts the consensus mechanism of Practical Byzantine Fault Tolerance (PBFT). Even if there are malicious nodes in the network, the integrity of the network can still be maintained through the consensus mechanism [19]. In order to maintain flexible data transmission and privacy, Fabric provides “channels” to isolate data that need to be protected, as well as the option to share private data with designated nodes [18].

Esposito et al. [20] proposed the smart city with identity management and access control, including Fabric technology, which may create many critical security problems and obstacles in the large-scale introduction into smart city facilities. The security and privacy of electronic medical records have always been necessary for patients and health care providers [12,13,21]. Wu et al. [21] used Hyperledger Fabric to implement smart contract control and electronic medical record access control. Wu et al. [21] believed that the healthcare system based on blockchain could improve the throughput of various data and reduce the delay of data transmission, thus improving the system’s robustness.

### 2.2. Decentralized Application

**Figure 1:**
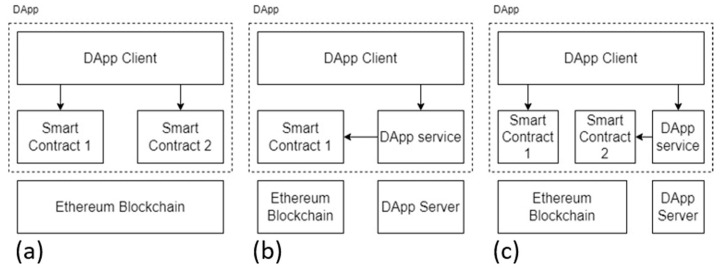
Three different architectures of distributed applications. (**a**) Direct Architecture; (**b**) Indirect Architecture; (**c**) Mixed Architecture.

Wu et al. [21] believe that based on blockchain Decentralized Application (DApp), all processes and materials should be processed and stored on the blockchain. Therefore, DApp also retains the technical characteristics of blockchain, including decentralization, data distribution on different nodes, non-tampering, and open source code. DApp does not require a centralized server to operate. Each device that downloads DApp is a part of the system and a node. When a single node fails, it ensures that the whole system will not collapse and will continue to operate normally. Wu et al. [21] divided DApp into three different structures, as shown in Figure 1, namely DApp of Direct Architecture (Figure 1a), where the user directly interacts with the smart contract. The Indirect Architecture DApp (Figure 1b) has a backend service running on a centralized server through which users interact with smart contracts. Furthermore, DApp with Mixed Architecture (Figure 1c) combines the previous two architectures in which the client interacts directly or indirectly with the smart contract through a back-end server.

### 2.3. Digital Certificate and Digital Passport

Xie et al. [14] developed a set of decentralized certificate systems providing blockchain certificate services. Eisenstadt et al. [8] developed a decentralized application and a decentralized server architecture for digital documentation and proof of vaccine administration in COVID-19. Abid et al. [16] used the InterPlanetary File System (IPFS) for public documentation of COVID-19 vaccine certificates and introduced the Know Your Customer (KYC) policy, safer interaction, and control for new users joining the system. Hasan et al. [22] established digital medical passports for people immunized against COVID-19, and uploaded records using IPFS and re-encryption proxies. Tsoi et al. [23] encrypted the vaccination data and distributed the vaccination data on the computers or mobile phones of hospitals, vaccine manufacturers, vaccine carriers, and other relevant participating organizations through blockchain technology. After the vaccination is completed, the vaccine passport will be automatically obtained.

All this related research on the digital certificate or DVP has a common drawback: they can only implement operation verification on the blockchain of their own country, but how to undertake cross-border digital passport verification has certain limitations and difficulties. In addition to the investigation of vaccine administration records, the COVID-19 digital vaccine architecture proposed in this study also enables cross-country data validation processes, which is rarely discussed in other digital vaccine architectures.

### 2.4. Identity Management

In any interconnected medical system and cross-organization, members in the system need to carry out secure and efficient authentication to avoid data leakage and ensure secure interconnection [24]. Digital dentity represents another avatar in the digital world, especially for users involved in the operation and browsing of sensitive data; the system must first conduct identity management for users [15]. Yazdinejad et al. [24] found that the current data sharing system requires a centralized data sharing architecture which requires a server or device to verify the user’s digital identity. The need for re-verification or multiple identities across organizations increases the risk of identity abuse to a certain extent [15].

**Figure 2:**
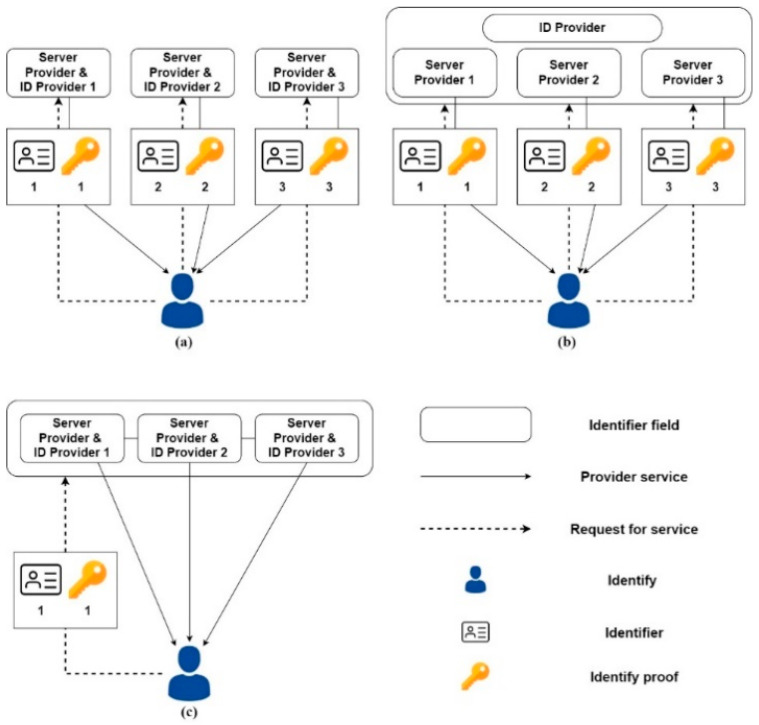
(**a**) Independent identity management architecture; (**b**) Centralized identity management architecture; (**c**) Federated identity management architecture.

Liu et al. [15] believe that identity management can be briefly divided into three architectures, as shown in Figure 2; the Independent Identity Management Architecture, Centralized Identity Management Architecture Architecture, and Federated Identity Management Architecture. The descriptions are as follows:

Independent Identity Management Architecture (Figure 2a): Each service provider holds its user identity data in this architecture. From another point of view, the identity data of different service providers are not interconnected, which belongs to a relatively simple structure.

Centralized identity management architecture (Figure 2b): Centralized identity management architecture registers the identity through a trusted certification authority to jointly identify the user’s identity in a single Trust domain. As the number of service providers increases, a user may need to register multiple identities in different places, which makes cross-trusted domain identity management and authentication difficult.

Federal identity management architecture (Figure 2c): the federal identity management architecture accommodates multiple service providers and the trusted domain under the scenario of authentication; one of the characteristics can be through the other service provider to the user identity authentication and identification, the status of the same users does not have to be registered, and they can still enjoy service in other places.

Since the development of DVPs requires cross-country verification, it is natural to adopt an international federal identity management framework to verify DVPs.

## 3. A Verification Framework of Digital COVID-19 Vaccine Passport

### 3.1. System Architecture and Process

**Figure 3:**
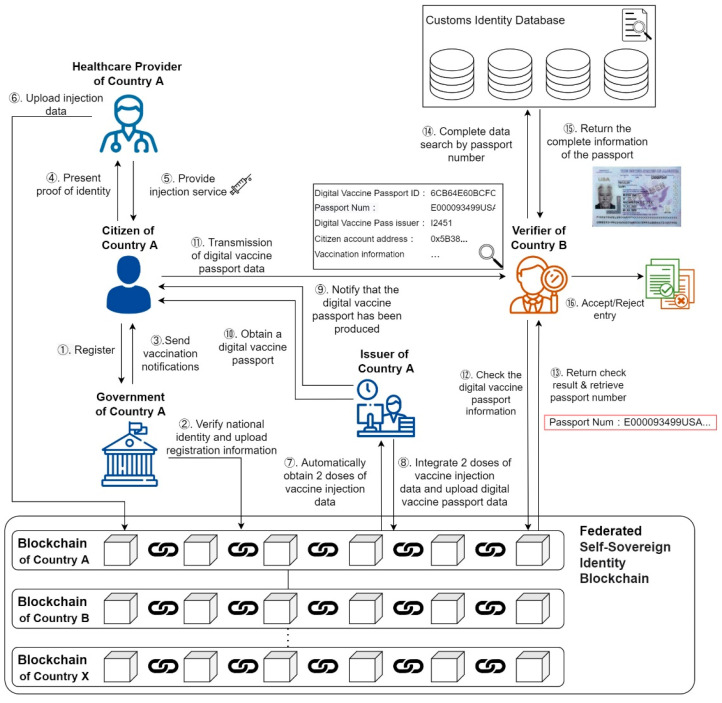
Scenario diagram of the Federal Hyperledger verification framework.

This section provides an international Federated Hyperledger verification framework based on a Federated Self-Sovereign Identity Blockchain. Figure 3 shows the architecture and process proposed in this study, including the issuance and verification of COVID-19 vaccine passports. The experimental environment of this study includes the operating system Ubuntu 20.04, and the software Docker Engine V20.10.13 and Hyperledger 2.2. The hardware used includes a 32 GB RAM CPU and a 1 TB hard disk. First, the citizen must register with the government of country A. After confirming the identity, the certificate can be uploaded to the country’s blockchain network (Steps 1 and 2). When the citizen receives vaccination notification to vaccination sites, they must show identification and DApp to do double verification (Steps 3 and 4), confirm the citizen being vaccinated, health care workers provide vaccination services, and the vaccination data uploaded to the citizen country blockchain network (Steps 5 and 6). Citizens who have received two doses of the COVID-19 vaccine will have their DVPs updated on the country’s blockchain network (Steps 7 and 8). The issuance of DVPs will be handled by specialized issuers of country A (Steps 9 and 10). When citizens enter another country, country B, they must present their DVP. Each DVP will have a unique QR code, and the foreign verifier of country B can perform DVP verification through Federated Self-Sovereign Identity Blockchain (Steps 11–13) and verify the traditional passport‘s identity. Finally, they can decide whether to allow or reject the entrance of the citizen from country A to country B. (Steps 14–16). More detailed steps will be introduced as follows.

**Figure 4:**
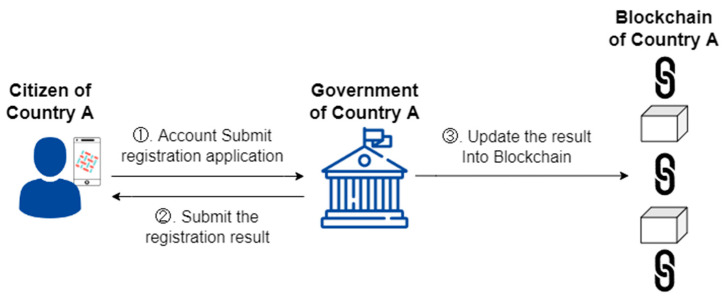
DApp account application scenario.

Previous studies have suggested that digital passport applicants must create their accounts before proceeding with subsequent operations and procedures [16,25]. Since someone may apply for an account with another’s identification, this study suggests that accounts be created through the government unit or local government health unit audit (Steps 1 and 2). After confirmed to be the own audit results into the blockchain (Step 3), the identity verification is completed through the DApp. Figure 4 shows the application process for a digital passport account.

**Figure 5:**
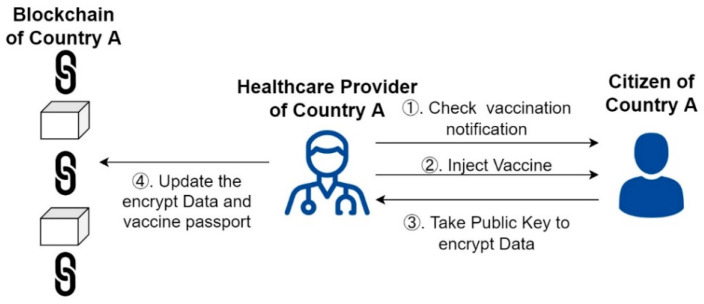
Scenario of the first dose of vaccine.

When citizens are registered, health units will notify eligible citizens to be vaccinated against COVID-19. The first dose of the vaccine is confirmed by DApp (Step 1). After the vaccination is completed (Step 2), the healthcare provider uses the public key to hash and encrypt the vaccination record of the citizens. After the encryption is completed, the data is uploaded to the blockchain network (Steps 3 and 4) to record the vaccination document of the first dose completely. The scenario of the first dose is shown in Figure 5.

**Figure 6:**
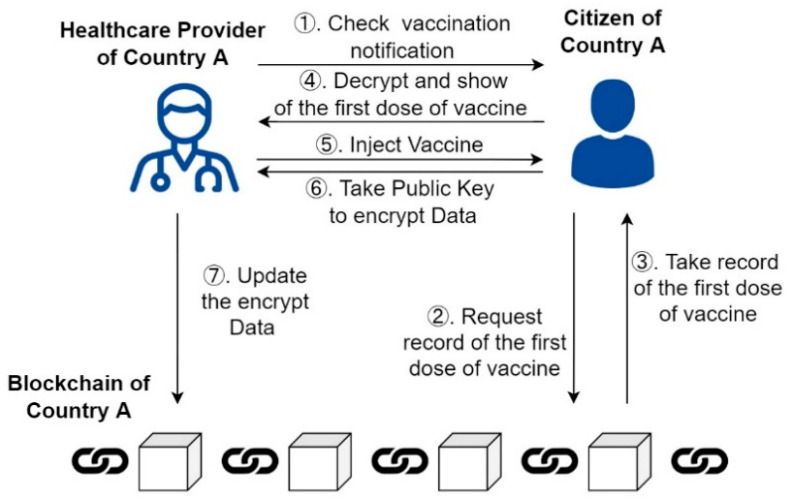
Scenario of the second dose of vaccine.

Figure 6 shows the scenario of the second dose of the vaccine. The identification process of the second dose of the vaccine is similar to that of the first dose of vaccine; citizens must first show the notification of vaccine administration to healthcare providers and obtain the record of the first dose of vaccine administration on the blockchain and show it to the healthcare provider for inspection (Steps 1–4). After being administered the second dose of the vaccine, the public key will be used to encrypt the record of the second dose of vaccine administered by the people (Steps 5 and 6). Furthermore, the record is uploaded to the blockchain network (Step 7).

**Figure 7:**
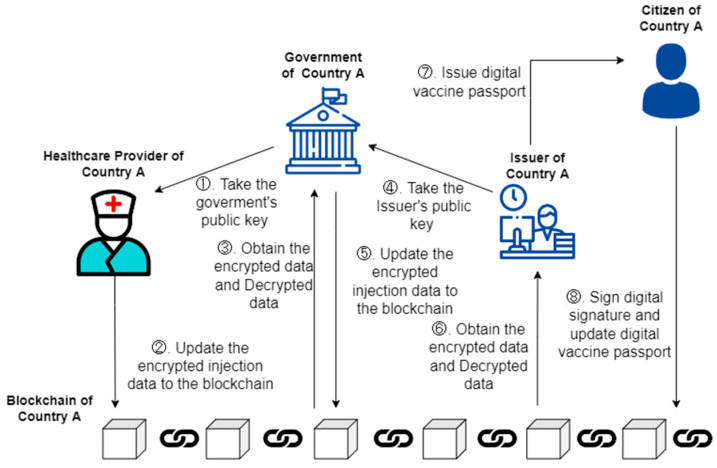
Digital COVID-19 Vaccine Passport access scenario.

Figure 7 shows the detailed steps obtained from the DVP of this study. After the vaccine administration of citizens is completed, in addition to using the public key of citizens to encrypt and upload the vaccine administration record. The healthcare provider is responsible for encrypting and uploading using the public keys of government entities (Steps 1–3) and the issuer is responsible for encrypting data from national vaccination records back to the blockchain using public keys (Steps 4 and 5). After checking the record for two doses of vaccine, the DVP issuing unit will send the draft of the DVP to the individual (Steps 6 and 7). After the person signs the DVP, the DVP will be uploaded to the blockchain (Step 8) to complete the citizen’s DVP issuance.

**Figure 8:**
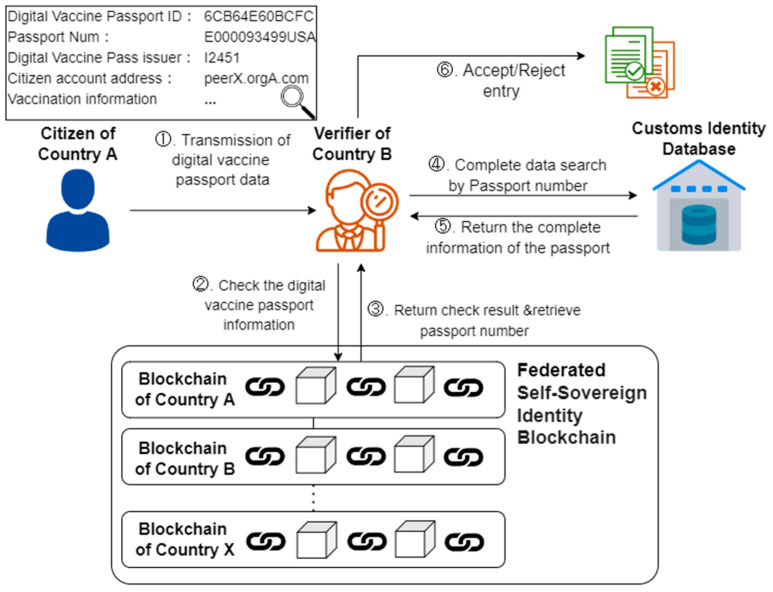
Digital COVID-19 vaccine passport verification scenario.

The verification process of this study refers to the study of Nabil et al. [25]. Figure 8 below shows the DVP verification scenario. After two doses of the vaccine are administered, citizens can obtain COVID-19 vaccine passports on the blockchain network using their private keys. When entering or leaving the country, it is displayed to the foreign Verifier (Step 1), which conducts a DVP verification through the Federated Self-Sovereign Identity Blockchain (Steps 2 and 3). DVPs contain the traditional passport number, which the verifier can use to confirm the identity (Steps 4 and 5) before deciding whether to admit citizens of other countries (Step 6). In the research process presented in this study, the essential risk to pay attention to is data privacy. How to protect personally identifiable information is critical. Mathew’s [26] work points out that using blockchain technology is much more secure than using a single tool. Many security researchers consider using blockchain to secure data because of its rigorous infrastructure, since every block of data shared will be hashed and connected to the next, making it impossible for a third party to modify it. Since only the communicating parties can read and manipulate the data, any stolen data will not be available and third parties will not be able to modify it. In the face of unauthorized access, blockchain technology can effectively secure access control to ensure user identification, authorization, and data transmission [26]. This study will also be a digital vaccine through blockchain technology for the main reasons.

### 3.2. Involved Actors

Considering that there are many roles involved in the system, the following roles are introduced in this study:

Citizen: Every citizen has a public and private key after installing the DApp and registering an account; however, the identity must be verified by the local government or health units, and the identity check and confirmation can be carried out before functions other than registration can be performed. After registration, DApp and official identification documents are required to conduct relevant interactive operations and exchanges, such as vaccine administration and PCR testing.

Healthcare Provider: This role is usually the local health authority. The local health bureau reviews the registration. After the application is approved, Healthcare Providers can upload vaccination data to the blockchain through the public keys of vaccinated citizens.

Verifier: Verify and confirm issued COVID-19 DVPs by scanning QR codes and checking digital signatures through registration review by local authorities or health units. The local government must authorize these verifiers to ensure safety and traceability. As long as the verifier is registered in the country of origin, he or she can use the federal identity management framework to access the blockchain network in the foreign country and perform digital passport data queries without needing additional identity registration.

Government: This role is usually that of the government health bureaus or the US Center for Disease Control (CDC). They are responsible for checking the identity of new users in the system, ensuring that other citizens’ real identities are not stolen or counterfeited. In addition, it can notify vaccination and issue COVID-19 DVPs and passports for PCR testing to the citizen. Citizens are eligible to be issued, and the smart contract is automatically submitted to the health unit.

Issuer: The issuer’s job is to review the issuance of DVPs and send the results back to the corresponding citizens when government health authorities send cases for review.

### 3.3. Chaincode

**Figure 9:**
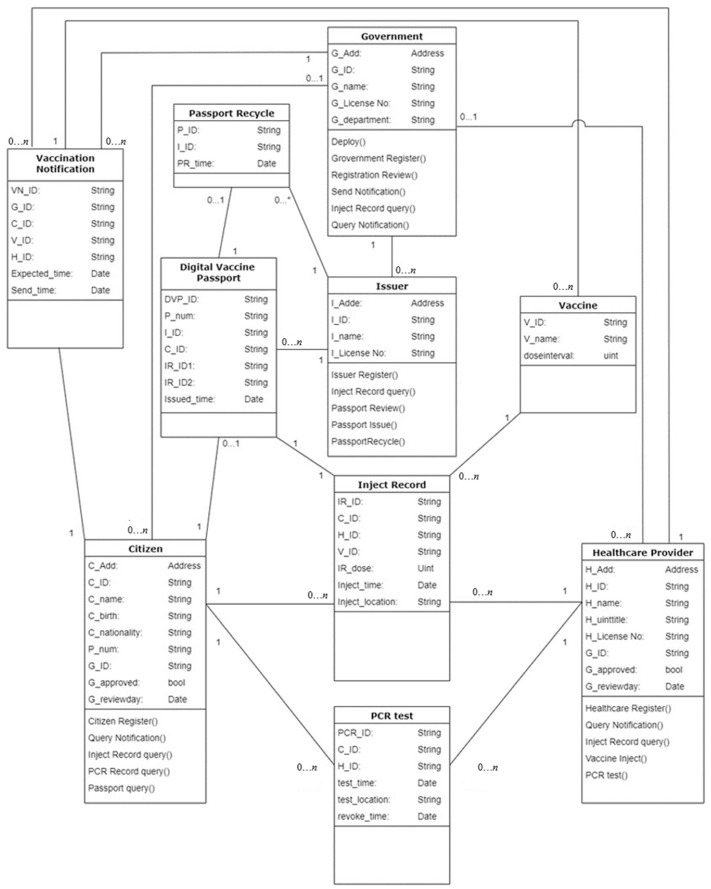
Entity relationship diagram.

This study developed a smart contract for distributing and verifying DVPs for handling COVID-19. The internal functions include account application, uploading vaccine injection records, PCR test records, and distributing DVPs. Users perform operations according to different requirements of roles. Figure 9 shows the entity relationship diagram of this study.

**Figure 10:**
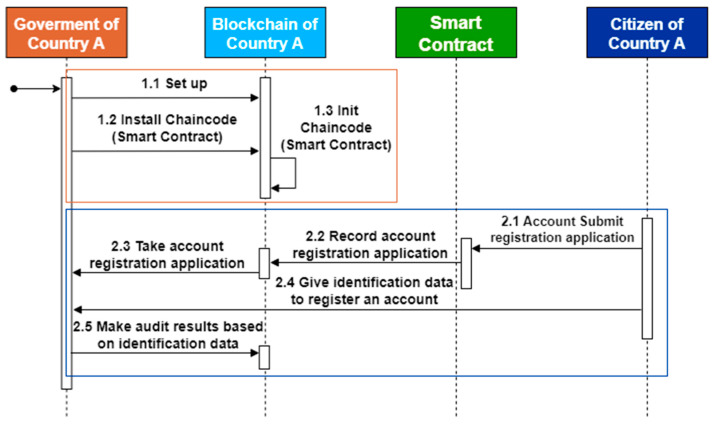
System setup and user registration diagram.

**Figure 11:**
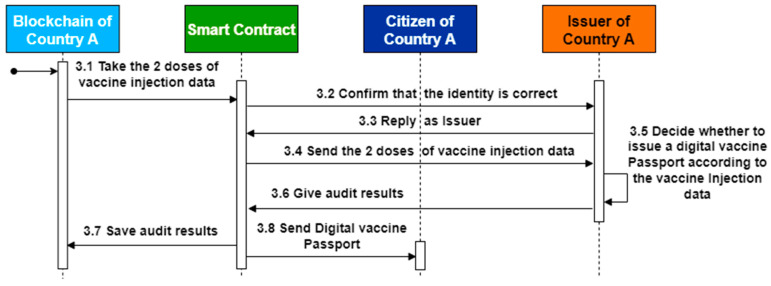
Sequence diagram of passport issuance of digital COVID-19 vaccine.

**Figure 12:**
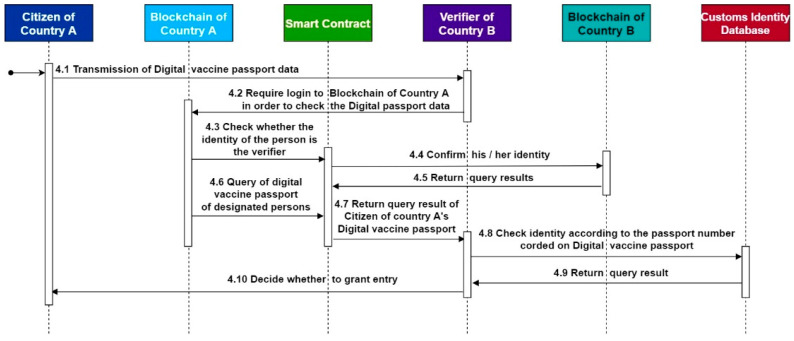
Digital COVID-19 vaccine passport verification sequence diagram.

In this study, smart contracts are used to realize system functions, and Hyperledger Fabric is used as the development environment of the research architecture. GO is the smart contract development language, and all contracts can be developed and tested through IBM Blockchain Platform (IBP) of Hyperledger Fabric. The development environment uses Microsoft’s cross-platform source code editor Visual Studio Code. In addition, the IBP extension module provided by IBM can be completed from the development, debugging, testing, and other functions simultaneously, so the development cost can be effectively reduced. The system algorithm of this architecture is introduced below, and the algorithm is written according to the sequential diagram in Figure 10, Figure 11 and Figure 12, and it is divided into algorithms 1–3 as follows.

Algorithm 1 shows the function of DVP registration from the citizen. This function allows citizens to submit their application for registration to the local government or health unit, and they must submit proof of identity for subsequent verification. A DVP registration can be completed after a professional civil servant completes the identity verification process using a smart contract.
**Algorithm 1** Citizen_Register**Input:** C_Add, C_ID, C_name, C_birth, C_nationlity**1****if** Check_Citizen(C_Add) is not exist **then****2**    Add CitizenInfo to CitizenaArrayList;**3**    Setting G_approved = false;**4**    str = “Application submitted.”;**5****else****6**    str = “Citizen has been registered”;**7****end if**

Algorithm 2 is exclusive to the issuer of the DVP, whose identity is created by the government. The DVPs will be automatically brought in by smart contracts to eligible citizens and submitted records, prior to delivery, records of administered vaccines will be checked and verified before DVPs are issued.
**Algorithm 2** Passport_Issue**Input:** DVP_ID, P_num, I_ID, C_ID, IR_ID1, IR_ID2, Issued_time**1****if** Inject_Record_query (IR_ID1) <> null && Inject_Record_query (IR_ID1) <> null **then****2** **if** Check_Issuer(I_ID) = Issuer ID **then****3**   Print query result from Inject_Record_query(IR_ID1);**4**   Print query result from Inject_Record_query(IR_ID2);**5****   if** info of inject record 1 is pass && info of inject record 2 is pass **then****6**    Add Digital Vaccine Passport Info to DVPArrayList;**7**    str = “Create Success.”;**8**   **else****9**    str = “Create Fail.”;**10**   **end if****11**
 
**else**
**12**   str = “You are not Issuer!!”;**13**
 
**end if**
**14****end if**

Algorithm 3 can verify that a traveler has a legitimate DVP at the time of international entry. The verification process involves the need for identification across countries and organizations. In the process of cross-organization verification, Hyperledger Fabric provides inter-organization identity verification and data verification in the form of an alliance chain. Both organizations only need to join a common alliance to verify data across organizations with data access permission without the hassle of registering an account in another organization’s blockchains.
**Algorithm 3** Passport_query**Input:** P_num, C_ID, DVP_ID**1****if** Passport_query(P_num) <> null && Passport_query(DVP_ID) <> null **then****2**  Print query result from Passport_query(P_num);**3**  **if** query result without question **then****4**   Print Passport query result from Customs Identity Database;**5**   **if** Passport query result without question **then****6**     Recorded as accept entry;**7****   else****8**     Recorded as Reject entry;**9****   end if****10**
 
**end if**
**11****end if**

## 4. Discussion

### 4.1. Performance Evaluation

Hyperledger Fabric as a platform is considered in this study and was used to perform an analysis of two world-state databases, CouchDB and LevelDB based on the suggestion of Sreenu et al. [27].

The performance evaluations are done using the Hyperledger Caliper benchmark tool used to measure the performance of blockchain solutions developed on Hyperledger. All benchmarks are driven at the maximum possible transactions per second (TPS) for a duration of 5 min. The benchmarks are comprised of Empty contracts, Create DVPs, Obtain DVP information, and Query DVP information. To evaluate the latency and overall throughput of the proposed system, this study uses the fixed chain code and deploys it under the channel of “Digital_passport” within the ‘CouchDB’ and ‘LevelDB’ networks, as suggested by IBM [28]. The Transaction Backlog is checked according to Caliper’s official recommendation that the test time is set at a transaction speed of 5 min and 350 TPS [29]. Several rounds of the same test process will be repeated to determine the results.

**Figure 13:**
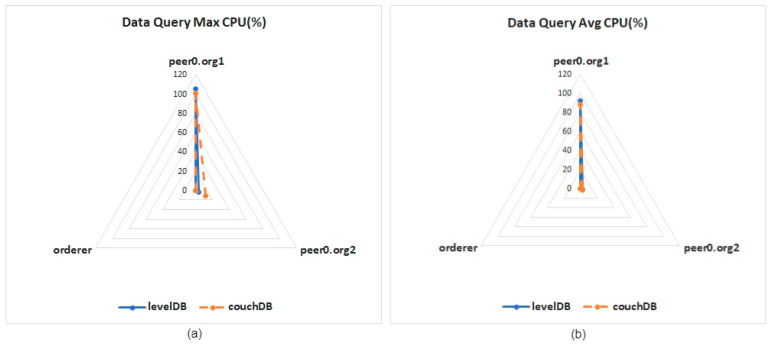
CPU usage for single data query: (**a**) maximum CPU usage, (**b**) average CPU usage.

The first test is a data query. Figure 13a shows the test result of calculating the maximum CPU usage. When node 0 (peer0.org1) of organization 1 uses CouchDB to query, the CPU usage required is 5% lower than that of LevelDB. Figure 13b shows the result of the average CPU usage in the data queries. The average usage of data queries for the two databases is quite similar.

**Figure 14:**
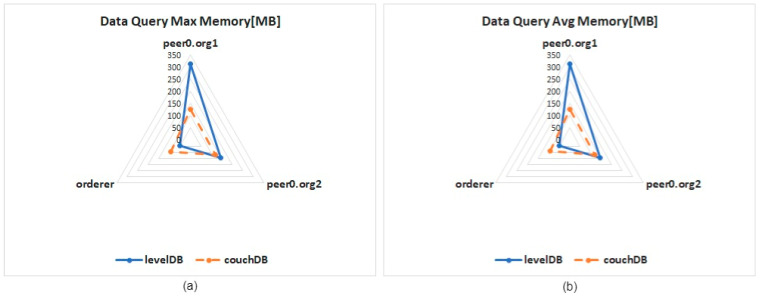
Memory usage for single data query: (**a**) maximum memory usage, (**b**) average memory usage.

Figure 14 shows the results of the memory test in the data query. When retrieving DVP data, LevelDB requires more memory for the data query. Taking node 0 (peer0.org1) of organization 1 as an example, when using LevelDB to query, it requires more memory than CouchDB, approximately 150 MB. When node 0 (peer0.org2) of organization 2 uses LevelDB to query, the memory requirement is slightly larger than that of CouchDB. The performance of the Orderer node is the opposite, the memory requirement of using LevelDB is lower than that of CouchDB, and the maximum difference is about 40 MB.

**Figure 15:**
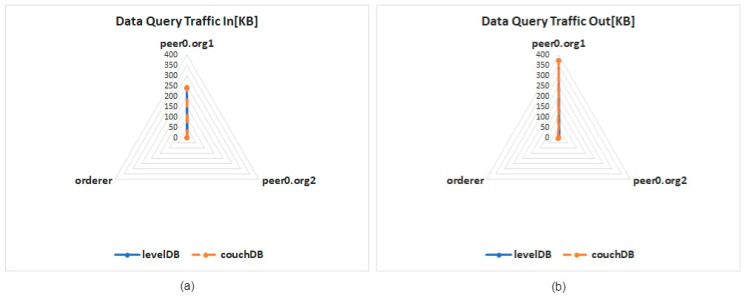
Network traffic for a single data query: (**a**) incoming packets, (**b**) outgoing packets.

Next, this study divides the network traffic into the inflow and outflow of the network. The network traffic test results are presented in Figure 15a,b. After the test, it is found that the inflow and outflow of two different databases is no obvious difference. Among the three nodes, when the data query operation is performed by node 0 (peer0.org1) of organization 1, the network usage is higher than that of the other two nodes, and the outflow is slightly higher than the inflow by about 130 KB.

**Figure 16:**
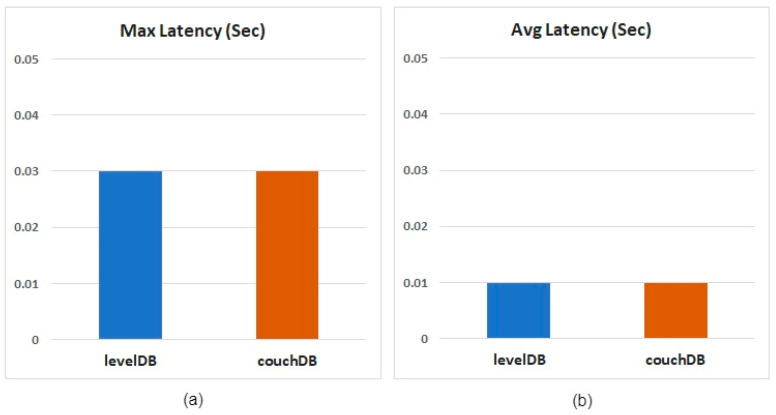
Latency results for single-data queries: (**a**) Maximum latency results, (**b**) Average latency results.

Finally, the latency result is shown in Figure 16. When the two databases are queried, the maximum delay of the query results does not exceed 0.03 s, and the average delay is 0.01 s. The time required from the start of the query to the return of the query results is quite short, which can be regarded as almost no delay.

### 4.2. Security Analysis

This section defines a federal Hyperledger verification framework for the DVP and analyzes the associated threats against the proposed structure. Finally, the advantages are compared with the results of other research.

In the proposed framework, when a citizen travels across national borders, authentication in other countries and information on the traveler’s vaccine administration is required, and federal identity authentication is used to confirm the citizen’s identity. To prevent data leakage or theft, secure communication is required during communication and data exchange. Although the blockchain network is highly secure and robust [22], if there is a loophole in the smart contract during the construction of the system, the system will be in danger. Therefore, the following security analysis for the proposed architecture is discussed.

Reentrancy: A reentrancy attack is a kind of network attack that is highly harmful and destructive to smart contracts. A reentrancy attack occurs when a function is called externally by another untrusted contract, untrusted contracts make recursive calls to functions within the original contract, potentially running out of money. Therefore, in this architecture, all functions are internally updated before being called by external contracts to prevent reentrant execution attacks.

Denial of Service (DoS) attacks: In DoS attacks, malicious actors attempt to block or interfere with the target computer by blocking the system servers so that normal users cannot access the network or computing resources. In distributed denial-of-service (DDOS) attacks, multiple attackers consume the system’s resources to disrupt its normal functions by sending unnecessary messages to the target device. In the proposed method, blockchain technology is resistant to such attacks [30], since this study adopts the architecture of Hyperledger Fabric and requires the license system for users or nodes to join. In other words, users who use the address of their account as a unique tag can track down and remove their access to the system even if an account they have joined intends to launch a DDOS attack. The architecture also cannot launch attacks anonymously, and the inability to launch DDOS attacks anonymously can reduce the possibility of being attacked.

Sybil Attack: In a Sybil Attack, the attacker disrupts the system by creating multiple fake identities and profiles; to address this issue, the architecture proposed in this study must be authenticated and investigated when digital identities are created, people who provide false information intending to join the system will be rejected, and they can only participate in the system operation with a unique identification or authentication when running on the system. In the system, the account address is used as the identification tag. As long as the malicious node cannot forge the identity, the possibility of interfering with the system operation will also be reduced [22].

Man-in-the-middle attack: A man-in-the-middle attack occurs when two parties believe they are communicating privately, but another hacker intercepts their communication; moreover, they impersonate the identity of both parties to intercept the communication content and tamper with the data [22], which is a dangerous thing to happen in the digital environment. This architecture takes blockchain as the main body for data exchange, which can prevent man-in-the-middle attacks. Any data sent on the chain will have a digital signature, which is generated through a user’s private key, which is difficult for others to copy and easy to verify, leaving no opportunity for middlemen to forge someone else’s identity.

Data Integrity and Privacy: In the blockchain environment, data generated in the system will be encrypted and stored in a decentralized manner, forming an immutable distributed ledger. Since each block is connected to the previous block, the original data must be modified simultaneously with the hash value of all subsequent data. Once the data is written into the system, it is difficult to change [21,29,30]. The architecture of this study uses blockchain technology, a public-private key encryption hash encryption algorithm, and the Hyperledger Fabric data control scheme. The use of a data isolation scheme known as a “channel” to keep data secure prevents unauthorized users from stealing the data, except the owner or authorized viewer of the data, and maintains adequate privacy for the data.

Scalability: Based on Sreenu et al. [27], the scalability of Hyperledger Fabric is promising, and according to the scenario described in this study, where vaccine passports are required will occur at the time of cross-border entry and border verification. Therefore, it is necessary to use the alliance chain to allow immigration officers from other countries to ban their databases for data retrieval or query, and the architecture based on Hyperledger Fabric supports the plug-and-play characteristics. In this system, 350 transactions are generated per second in the environment simulated by Caliper’s benchmark and the research architecture. Network latency also has good performance and can provide fast query service.

Table 1 shows the security analysis and comparison of other research in the past, including the discussion of reentrancy, denial of service attacks, Sybil attacks, and man-in-the-middle attacks [8,11,14,16,22,31]. Meier et al. [31] lack of in-depth discussion on the integrity of data. Yazdinejad et al. [24] lack analysis of reentrancy, Sybil attacks, and man-in-the-middle attacks. Hasan et al. [22] lack discussion of Sybil attacks. In conclusion, this study establishes the excellence of system security in the proposed architecture.

Table 2 shows the comparison of Hyperledger Fabric architecture in digital vaccine passports and other research in the past, including the Vaccination record, Vaccination certificate, Consortium, and FIM. Tsoi et al. [23] proposed a possible framework of DVP without implementing a consortium. Wilford et al. [32] only present regulatory risk and policy challenges of vaccine passports. Rani et al. [33] and Nabil et al. [25] lack consortium and FIM implementation in the blockchain. Cao et al. [34] have a blockchain of consortium idea but lack FIM implementation. In conclusion, this study establishes the excellence of cross-country data validation processes in digital vaccine passports via FIM.

## 5. Conclusions

Due to the outbreak of COVID-19, in order to prevent its transmission, all countries need to provide information on vaccine administration that can be verified by electronic records which will inhibit the spread of COVID-19 and significantly improve the effectiveness of epidemic prevention. The architecture proposed in this study can verify DVPs across countries and organizations and protect personal data privacy under the encryption technology and identity the control scheme provided by Hyperledger Fabric, which prevents unauthorized personnel from being protected from viewing other people’s data. By establishing an international, federal Hyperledger verification framework with privacy protection, anti-tampering, anti-identity theft, and proof of vaccine administration, citizens of all countries can upload their vaccination certificates to the blockchain, and border agents of other countries can check their DVPs through the Alliance’s blockchain network when entering and exiting the border. This study takes Hyperledger Fabric as the proposed architecture, and the security analysis results show that the proposed architecture has little potential security vulnerabilities or significant risks. The experimental results show that under the pressure of executing 350 transactions per second, the nodes need low computation cost and low network latency for cross-organization data queries, and the future scalability is worthy of expectation.

The urgency for DVPs is somewhat lower than in the past, as some countries are gradually liberalizing their restrictions on pandemic control and border verification standards. However, the framework proposed in this study can still provide an example of the exact cross-country certification needs in the future. For example, based on this framework, it can be converted into a blockchain network of the transnational alliance of criminal records. After conversion, it can upload domestic criminal records, and when a cross-country criminal or criminal records search is required, a cross-country criminal records database search is possible.

**Figure 17:**
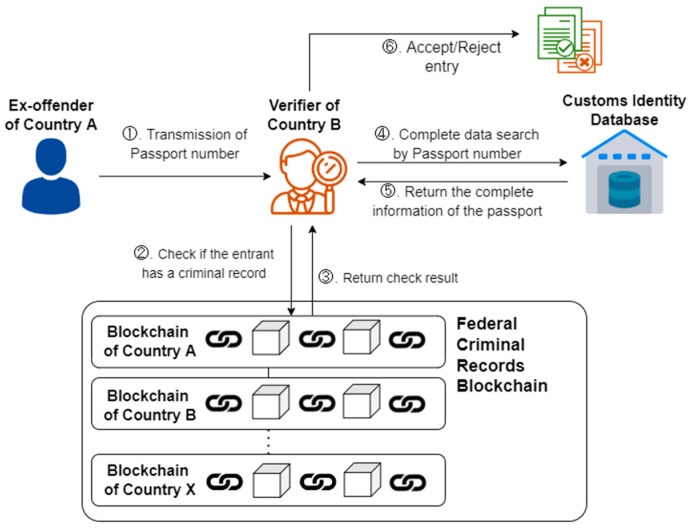
Transnational criminal record query scenario.

Figure 17 shows the transnational investigation situation of criminal records. Bueger and Edmunds [35] pointed out that ex-offenders and offenders may escape from the country due to a lack of information sharing. If the consortium blockchain architecture proposed in this study is used for checking, ex-offenders will not be able to hide their past criminal records in any country. In short, the architecture proposed in this study is worthy of further exploration and application in future research. However, the international FIM process of acceptance, approval and certification from each country/jurisdiction are not easy to accomplish. In terms of international implementation, this issue has to be carefully considered.

This architecture can be extended to areas such as health care, but with this comes the issue of cost. Health care systems worldwide face challenges that often lead to increased costs. When stakeholders are unwilling to cooperate with each other, the consequences are rising health costs and reduced health outcomes. Through the integration of blockchain technology, it has a great relationship with scale and storage space. According to Chukwu & Garg [17], if 50 random patients are constructed into the AWS cloud through the Ethereum technology of blockchain and stored for nine days, it is found that each patient must spend $283. The total stored data is also equivalent to 2.123 megabits, and the cost issue cannot be ignored.

In addition, more optimization work related to the account registration and verification process can be considered in the future. The current proposed structure cannot find an identity certificate issued by the country or government agency for the time being. It is better to use it as the only identification method. There may be a better way to link physical identification to digital vaccine certification; therefore, this study believes that in the future, we can find other better ways to distribute DVPs and identification certificates that protect both privacy and information security. At the same time, if there is an outbreak of similar large-scale infection in the future, it can be deployed according to the framework of this study to facilitate the verification of future border epidemic prevention or vaccine application records.

## Figures and Tables

**Table 1:** Security analysis of existing studies.

Author	Reentrancy	DoS	Sybil Attack	Man-in-the-Middle Attack	Data Integrity	Data Privacy
Yazdinejad et al. [24]	-	ˇ	-	-	ˇ	ˇ
Eisenstadt et al. [8]	-	-	-	-	ˇ	ˇ
Abid et al. [16]	-	-	-	-	ˇ	ˇ
Xie et al. [14]	-	-	-	-	ˇ	ˇ
Xu et al. [11]	-	-	-	-	ˇ	ˇ
Hasan et al. [22]	ˇ	ˇ	-	ˇ	ˇ	ˇ
Meier et al. [31]	-	-	-	-	-	ˇ
Our study	ˇ	ˇ	ˇ	ˇ	ˇ	ˇ

ˇ: stands for done; -: stands for uncertainty.

**Table 2:** Comparison of Hyperledger Fabric researches in digital vaccine passport.

Author	Hyperledger Fabric	Vaccination Record	Vaccination Certificate	Consortium	FIM
Wilford et al. [32]	ˇ	-	-	-	-
Tsoi et al. [23]	-	ˇ	ˇ	-	-
Rani et al. [33]	-	ˇ	ˇ	-	-
Nabil et al. [25]	ˇ	ˇ	ˇ	-	-
Cao et al. [35]	ˇ	ˇ	ˇ	ˇ	-
Our study	ˇ	ˇ	ˇ	ˇ	ˇ

ˇ: stands for done; -: stands for uncertainty.
